# A Vulnerable Point

**Published:** 1875-12

**Authors:** 


A VULNERABLE POINT.—Anybody who happens to ride in one of our street cars on a bitterly cold day must be not a little amused at the high fur collars, fur caps, fur ear-warmers, and woolen bandages which the average passenger employs to protect his neck, head, and ears, from the frost. But it is rather odd that the instinct of self preservation should be manifested for the head alone, and that no care should be taken of the feet, considering that they stand most in need of attention. If accurate statistics could be obtained it would be found that a large per centage of the deaths from consumption are attributable to carelessness in the manner of protecting the feet. A young man who died recently in a neighboring city, in giving a friend a description of his disease, said he was "as strong as a horse" until a certain night, when in riding to his home from a ball in light-soled shoes his feet became "as cold as stones or ice." After that he had never been well again. It would take a long time to count the number of those yet living who date the beginning of their loss of health to some such circumstance; but it is doubtful whether many of their friends have taken a lesson from their sufferings.

---

A SET BACK—"Jennie, you're my sweetheart," said a nine-year-old suitor, as he sat alone with his heart's idol, recently.

"How can I be your sweetheart," asked the little Miss, "when I am thirteen years old, and you only nine?"

"Are you thirteen?"

"Of course, I am."

"Well," answered the juvenile beau, after reflecting a little, "I'd a been thirteen, too, if I hadn't been sick so much when I was little."

